# Atlas of the Diseases of the Skin

**Published:** 1878-08

**Authors:** 


*Atlas of the Diseases of the Skin.* Part I. By Balmanno Squire, M. D., Surgeon to the British Hospital for Diseases of the Skin. London: J. & A. Churchill. Demy Octavo. pp. 89. With four lithographic plates.

This is the first of a series of parts the author intends to publish from time to time on diseases of the skin. It is his intention to represent in the lithographic plates accompanying the text, the life size appearance of disease without attempting to show any greater extent of surface than an octavo page will allow. The subjects considered in the first number are nævus and psoriasis. The authors method of treatment of nævus by repeated scarification is thoroughly presented and his success has been gratifying. Other methods are given but his own is doubtless preferable in ordinary cases. Ten years ago or more, distinguised authors published an atlas upon skin diseases and a desirable contribution to the literature of this department may be looked for in the new series he has now undertaken.

